# Behavior of polyether-ether-ketone (PEEK) in prostheses on dental implants. A review

**DOI:** 10.4317/jced.58102

**Published:** 2021-05-01

**Authors:** Natalia Blanch-Martínez, Santiago Arias-Herrera, Amparo Martínez-González

**Affiliations:** 1 Universidad Europea de Valencia. Faculty of Health Sciences. Department of Dentistry; 2 Clinical and Applied in Implant-Prosthetics (ICAI) Research Group, Universidad Europea de Valencia. Faculty of Health Sciences. Department of Dentistry

## Abstract

**Background:**

The development of new and innovative materials such as high performance polymers (PEEK) opens a wide therapeutic range in implant prostheses. They are presented as alternative materials to metal and zirconium alloys in the manufacture of structures and attachments for implant prostheses and fixed and removable dental substitutes. The objective of this review is to know the characteristics of this material and thus assess its advantages and disadvantages in its possible applications in prostheses on dental implants.

**Material and Methods:**

A bibliographic search was carried out through the PubMed and Scopus search engines, of articles published from 2007 to 2020, excluding all articles in which PEEK was used for tooth prostheses. The data on the sufficiency of the PEEK material were organized according to its chemical, physical and mechanical properties.

**Results:**

148 articles were found in the databases using as keywords; Polyetheretherketone; PEEK; BioHPP; healing abutments; dental prostheses; dental prosthodontics; Full-arch rehabilitation; fixed implant prosthodontics; implant-retained prostheses; implant prostheses.

**Conclusions:**

It is concluded that PEEK offers greater lightness, good aesthetics, biocompatibility, and an elastic modulus more similar to bone than other materials commonly used in implant prostheses; however, it presents a higher risk of fracture and abrasion. More long-term clinical studies will be necessary to advise its use in implant prostheses.

** Key words:**Polyetheretherketone, PEEK, BioHPP, healing abutments, dental prostheses, dental prosthodontics,Full-arch rehabilitation, fixed implant prosthodontics, implant-retained prostheses, implant prostheses.

## Introduction

One of the challenges of modern dentistry is to improve the biomechanical and biocompatible properties of the materials used for implant treatments (1). Metals, ceramics, and polymers stand out among the materials that are going to be used to make the suprastructures and different attachments on dental implants. The recent great demand for metal-free materials to be used in the oral environment due to their corrosion and ion release problems, is contributing to the development of the latter (1).

Polymers are materials made up of macromolecules; These in turn are made up of smaller molecules, monomers, which can form linear or racemic chains. According to this association they will have some properties or others (2).

In general, polymers have lower elastic moduli and experience greater elongation to fracture than other types of biomaterials. Compared to bone, most polymers have lower elastic moduli, of magnitudes close to those of soft tissues (2). They are thermal and electrical insulators when used in high molecular weight forms, without plasticizers and are relatively resistant to biodegradation (1).

Among the most inert polymeric biomaterials, it is worth mentioning polyetherfluorothylene (PTFE), polyethylene terephthalate (PET), polymethylmethacrylate (PMMA), polyetheretherketone (PEEK) and ceramic-filled polyetheretherketone (BioHPP) (3).

PEEK is a partially crystalline polymer widely spread in the industrial world that, little by little, has been introduced into the world of biomedicine. It was patented in 1981 as implantation material and accepted in 1990 by the FDA (Food and Drug Administration, USA), especially in the areas of Orthopedics and Traumatology, but also in Neurosurgery(3). In 1988 the PEEK material was approved for oral application in dentistry, being in 2011 when it began to use the material in the implantology field; it began to be used quite effectively in temporary and permanent implant abutments and healing screws. But it is increasingly being used in overdentures and hybrids as well as screw-retained bridges in implant prostheses. Its versatility, biocompatibility and biomechanical properties make this material a promising substitute for alloys in the mouth (3).

The objective of this narrative review is to know the characteristics of this material and thus assess its advantages and disadvantages in its possible applications in prostheses on dental implants.

## Material and Methods

The authors performed an initial electronic research in MEDLINE via Pub-Med and Cochrane Central Register of Controlled Trials until January 2020. The literature search was conducted using the combinations of the following Medical Subject Heading (MeSH) and text words: Peek AND (dental prostheses) ,Peek AND (dentalprosthodontics) , Peek AND (Full-arch rehabilitation), Peek AND (fixed implant prosthodontics) , Peek AND (implant-retained prostheses), Polyetheretherketone AND (implant prostheses) (fixed implant prostheses) AND (metal-free) AND peek ,Peek AND (implant-supported fixed dental prostheses) The inclusion criteria in the selection of the articles were articles published from 2012 to 2020, both included, *in vitro* studies where characteristics, properties and applications of PEEK were analyzed. Were excluded studies where PEEK is used, for other treatments or for the manufacture of dental implants and studies on PEEK in natural teeth.

The titles and abstracts of all the “potential articles” to be included in this work were examined, obtaining the full articles of those that we consider relevant. Once the full articles had been read and analyzed, only those that met the inclusion criteria were included. At this time, so that no work of interest on the subject escapes our selection, we search the reference lists of all the selected texts as well as the “related articles” indicated by the databases.

## Results

**Figure 1:**
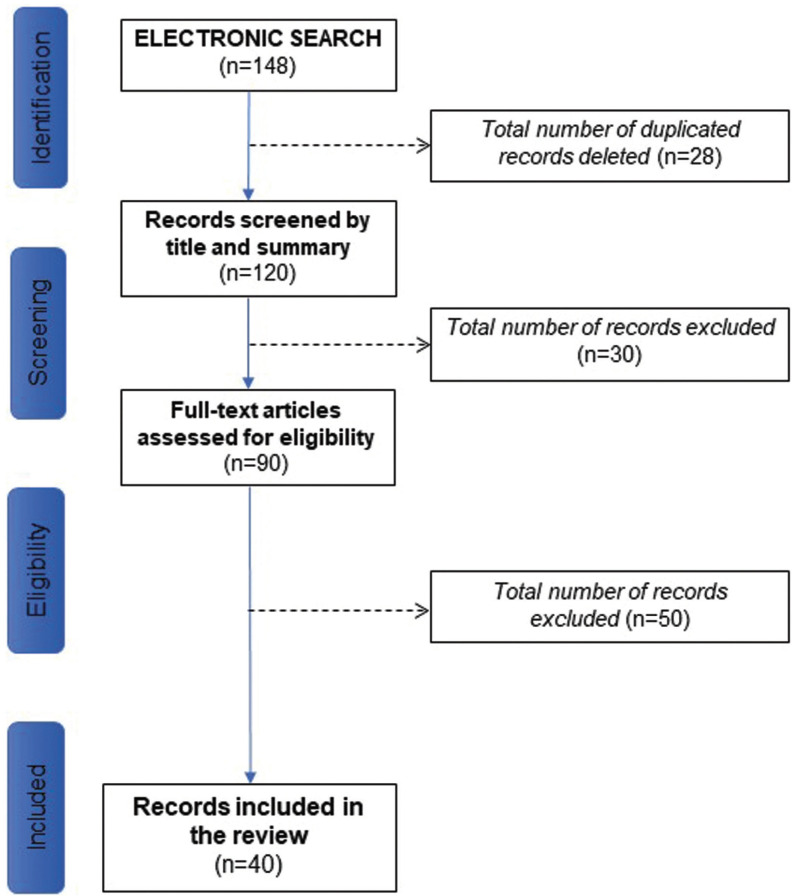
Flow-chart.

In the search, a total of 148 articles were detected from the electronic database MEDLINE (Pubmed), 52 through a manual search in the journals with the highest impact index. After eliminating duplicates, a total of 120 studies were identified, 50 were excluded after screening by title and abstract. After screening the full text of the remaining, 70 articles, 30 were excluded because they did not meet the inclusion criteria. Finally, 40 articles were included in the review (Fig. 1).

## Discussion

-General characteristics of peek

The structure of this polymer is made up of polyaromatic ketones, which give it great stability at very high temperatures, above 3000ºC and greater resistance than many metals. It is hydrophobic and insoluble in any solvent except sulfuric acid at a high concentration (2). It is not susceptible to hydrolysis, this makes it a better material than, for example, carbon fiber, which it is. This material also has high radiation stability and is radiolucent. It is a light material and has great resistance to deformation and very good biomechanical properties, compared to other materials used up to now, such as Titanium and Zirconium (2). It presents an excellent polish, therefore, little propensity to retain bacterial plaque (9).

Its color is white, so it has a good aesthetic and the possibility of customization. Its insolubility in water makes it a biocompatible material, ideal for allergic patients. It is also a material that does not present corrosion, therefore it does not degrade due to saliva, acid pH, food or drinks, bacterial plaque, etc. (9). It also does not present degeneration due to aging, it is very resistant to gamma and X radiation, it feels like a chemically sTable material (2).

Polyether-ether-ketone can be combined with other materials such as carbon fiber or ceramic particles (BioHPP), thus improving some of its properties. PEEK reinforced with carbon fiber is even comparable, with respect to elastic modulus, with the cortical bone and dentin, thus reducing the stress that can be caused to the bone and avoiding resorption and future damage (5).

-Presentation of the peek

PEEK can be found in the form of granules, powder or ultra-fine powder, depending on the molding technique used.

A. Injection molding: it consists of injecting a polymer in a molten state into a closed, cold pressure mold through a small hole called a gate, filling all the space and adopting the shape of the desired part; the use of granules is recommended.

B. Extrusion molding: use helical screw conveyor; the polymer is transported from the hopper, through the heating chamber, to the discharge mouth; the use of powder is recommended.

C. Compression molding: it is a part-shaping process in which the polymer introduced into an open mold to which pressure and heat are then applied so that it takes the form of a mold; the use of ultra-fine powder is recommended (1).

**Figure 2:**
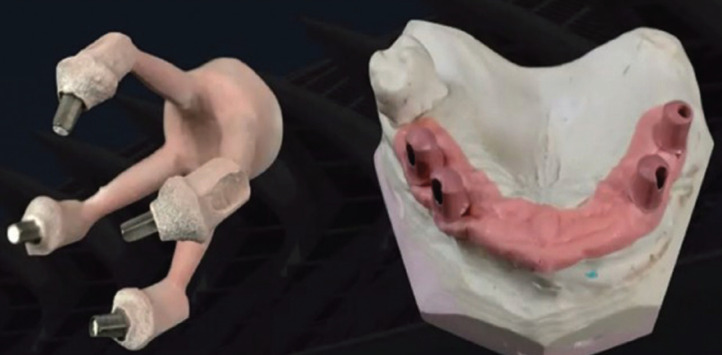
PEEK structure designed by computer.

For its use in dental prostheses, two manufacturing procedures are described, the Injection Procedure and the CAD-CAM procedure. In the latter, from some PEEK blocks, using a milling machine, the structure previously designed by computer is manufactured (3) (Fig. 2).

**Figure 3:**
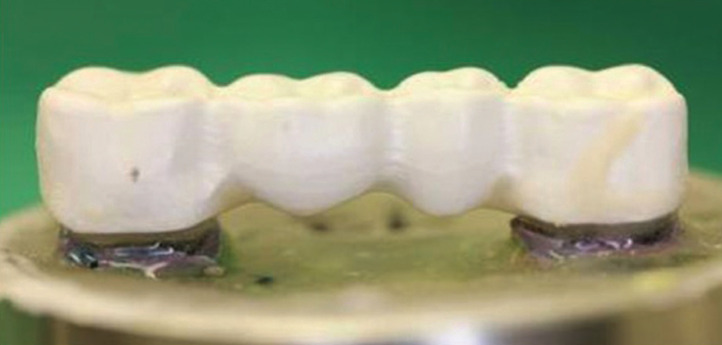
PEEK trasnepithelials.

It is noteworthy that materials such as polymethylmethacrylates (PMMA) are easy to inject, but Peek requires a special vacuum pressing system (Bredent®, Bredent GmbH & Co. KG- Weissenhorner Str. 2.89250 Senden-Germany) (2). On the other hand, the CAD-CAM method, milling the industrially elaborated material in disk molds, allows working with a homogeneity of the material, quality and unalterable characteristics (Juvora®, from Invibio®, Peek-Optima®, Bredent GmbH & Co. KG- Weissenhorner Str. 2.89250 Senden-Germany) (3), (Fig. 3).

Once the PEEK structure is finished, an important role to take into account is the preparation of the surface of the same, to add the adhesive and the composite or the coating resin. Condensation and moisture-free layering are vital for good adhesion (10). Finally, the prosthesis is perfectly polished with the appropriate discs, which gives a surface smooth little adherent for bacterial plaque.

There are currently several classes of PEEK on the dental market.

**Figure 4:**
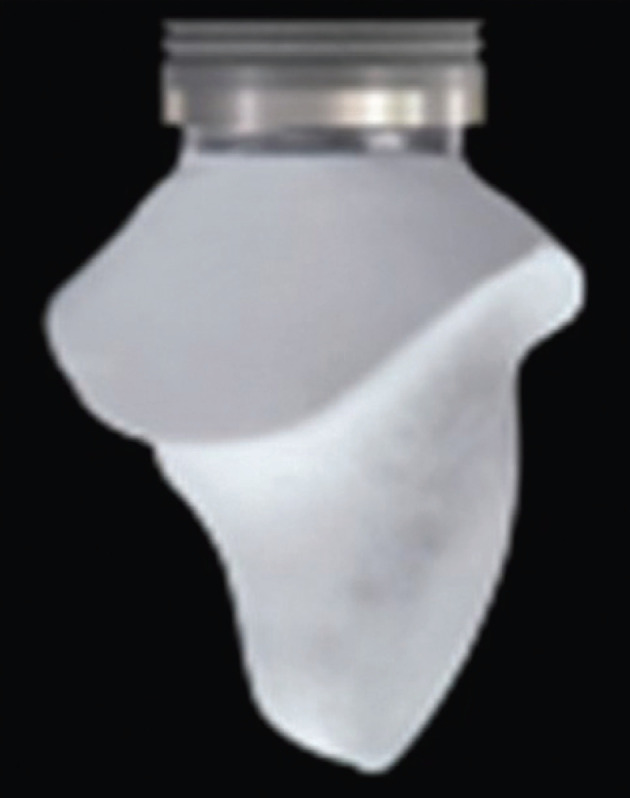
Peek reinforced with ceramic.

1. Natur Peek: its producer is Juvora, from Invibio®, and it is marketed through Schütz (Innoblanc, Goldquadrat). It comes in grayish and brown colors.

2. Peek with color: its producer is Evonik and it is marketed through Bredent (Denseo, Merz). Contains aluminum oxide and color pigments. It is whitish or tan in color.

3. BioHPP: based on PEEK and the presence of ceramic molecules (aluminum oxide and zirconium oxide (Bredent®). This Peek is reinforced with ceramic, which has improved its properties of the base PEEK (3) (Fig. 4).

-The peek in implantology

Knowledge of the mechanical characteristics of PEEK is important to deduce the indications for which it can be applied. The following characteristics are decisive: the modulus of elasticity and the maximum resistance to breakage, the strength of the bond to aesthetic coating materials, as well as the polishing properties. All this will have an impact on the biological and mechanical parameters of the treatments.

-Elastic modulus and resistance to fracture

According to Hendrik J. *et al* the modulus of elasticity of PEEK is around 4,000 MPa, a value that is very close to that of human bone(4). This dampens the chewing forces, especially in the case of subsequent restorations on implants, regarding its transmission to the peri-implant environment. In addition, it also confers adequate resistance to fracture. In *in vitro* studies analyzed such as that of Nazari V. *et al.* where up to 1200 Nw was applied to PEEK crowns, Zirconium and Chromium-Cobalt, on two Titanium implants, it was observed that when applying said vertical load in a three-piece bridge made of PEEK, there was no fracture of the material itself, with which compared to a masticatory force of maximum 500 Nw in a human denture, represents a sufficient safety potential, compared to other materials (5).

But nevertheless, according to Hang-ying J. *et al.* this materialin pure state,it has a low resistance to bending fatigue, thus limiting its application. Pure PEEK has been used provisionally in prostheses, until another component, zirconium oxide, has been introduced, forming the so-called BioHPP, to make both abutment abutments and the suprastructures of overdentures or hybrids themselves (8). They carried out an *et al.* study of continuous loading, comparing the resistance that the abutments of pure titanium unitary prostheses had when coated with resin, with respect to abutments of BioHPP coated with resin. They found that BioHPP could withstand loads of up to 1,518 N until the abutment itself fractured.

At work of Preis V. *et al.* wanted to compare flexural strength through a study comparative *in vitro* of the characteristics of this material (BioHPP) with respect to other types of materials manufactured so far, including Zirconium coated lithium disilicate. Observing that when applying different forces in different directions, any of the materials resisted the simulated forces without reaching fracture (10).

-Bond strength to coating materials

It is worth noting the bond strength to other materials that this polymer presents, since it is decisive that the structure can be coated with all the usual coating composites. A study by Hang-ying J. *et al.* compared the bond strength of this material and its modified variant (BioHPP), with respect to the Cr-Co alloy and metal-ceramic, resulting in better adhesion of the PEEK material (8). Polymers, such as BioHPP, are all opaque and are veneered with veneering composites for aesthetic reasons. The surfaces to be coated would undergo different pre-treatments to increase the strength of the bond. In this study, the bond was assessed by adhering the structure material with coating materials from different common brands on the market depending on the conditioning(8). It was concluded that BioHPP together with some special coating resins had higher adhesion ranges and could withstand loads of up to 1500N without undergoing any type of fracture (Fig. 2).

-Bacterial plaque retention

Resistance to bacterial plaque is another of the characteristics that PEEK offers compared to other materials, as argued by Wang L. *et al.* Where they apply to different PEEK structures. particles of nano-fluorohydroxyapatite, which makes it resistant to the adhesion of the bacterial plaque of the cavity ora. Due to the nature of the PEEK surface and its low roughness of 0.018 Nm Ra, soft tissue irritations do not occur (9).

-Use of PEEK in implant abutments.

Regarding the type of prosthesis most used with this new material and after reviewing different articles *in vivo*, we see that the type of prosthesis most used are structures for overdentures and hybrids in totally edentulous patients both in the upper jaw and in jaw we also observe studies where the operated patients are placed immediately, provisional prostheses of single crowns and maximum sections of three pieces, so we could suggest that the type of prosthesis most used for this material would be provisional and immediate loading (3).

Another point to be discussed is the use of PEEK as a material for the manufacture of abutment attachments. Until now, it has been seen that titanium is the most resistant material for the manufacture of abutments when it comes to supporting loads, but in recent years PEEK has been introduced for its design.

Hendrik J. *et al.* conducted an *in vitro* study comparing three classes of abutments, Rn synOcta Meso (PEEK), Rn synOcta Titanium Post, Engaging Nobel RpLRP (Zirconium), where these abutments received a maximum load of 2,000N and observed that there was noa significant difference between the Titanium abutment and PEEK, with the ceramic abutment being the least resistant (4). On the other hand, Tekin S. *et al.*, compared the resistance to fracture in an oblique and vertical load, between screws for titanium transepithelials, with respect to screws for PEEK transepithelials, observing lower tensions generated to the abutment itself in the PEEK screws, with respect to the titanium screws (11). Another study by Schwitalla AD *et al.* defined as an advantage, the easy recoverability of PEEK screws, compared to those of titanium, in crowns on implants, when there was some type of fracture (12) (Fig. 3).

-Biological and mechanical parameters of the treatments analyzed

When using a new material, it is important to analyze *in vivo* studies with a certain follow-up time, the results that we have obtained after reading several bibliographic reviews, is that from a biological point of view such as peri-implantitis and implant survival, as well as possible problems prosthetic mechanics, PEEK is an ideal material to be used in the world of implant prosthetics, better managing the stresses to the surrounding bone (9), and to the different attachments that support the rehabilitation itself (11), (Table 1,  Table 1 cont.).

**Table 1:**
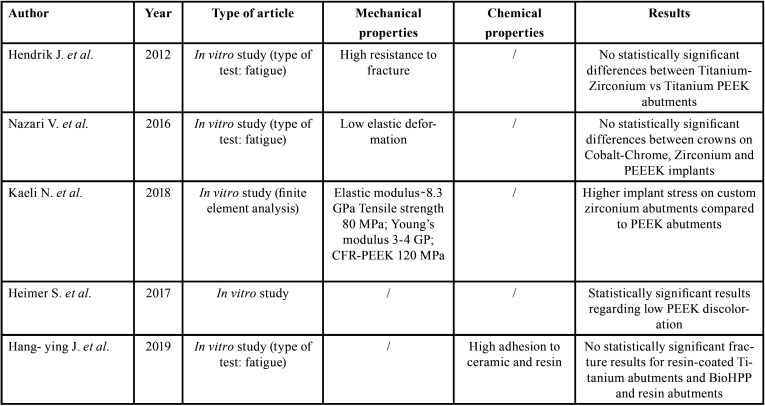
Included studies.

**Table 1 cont.**
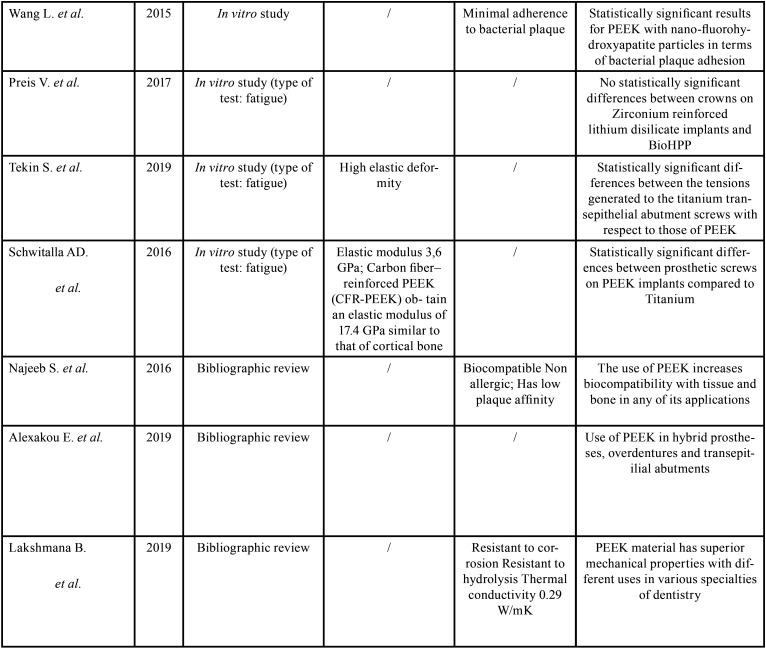
Included studies.

## Conclusions

1. Of all the characteristics of PEEK, the ones that represent an advantage for its use in prostheses on implants is its low elastic modulus, similar to that of bone, its low hardness, which will not cause an abrasion of the opposing tooth as occurs with the ceramic, its good polishing with which there will be less adhesion of bacterial plaque and finally, its good adhesion with coating materials, which guarantees better resistance to detachment or chipping. On the contrary, it has some characteristics that can be inconvenient, such as the possibility of deformation under stress and certain solubility in water and water absorption of the coating resins, since it will be immersed in a humid environment such as the oral environment.

2. From the reviews of articles reviewed, we conclude that this material is used for implants, in totally edentulous patients for the fabrication of the suprastructures of hybrid prostheses and overdentures, as well as in partially edentulous patients for the fabrication of abutments and crowns on implants. observing especially its use for abutments, screws, and crowns on implants.

3. We cannot conclude anything on the survival of prostheses made with PEEK after a time in the mouth, given that we lack a sufficient number of studies with scientific evidence and a follow-up in time to support it.
